# Antiangiogenic Polyketides from *Peperomia dindygulensis *Miq

**DOI:** 10.3390/molecules17044474

**Published:** 2012-04-13

**Authors:** Qi-Wei Wang, De-Hong Yu, Meng-Gan Lin, Mei Zhao, Wen-Jun Zhu, Qin Lu, Gui-Xiu Li, Chao Wang, Yi-Fang Yang, Xue-Mei Qin, Chao Fang, Hong-Zhuan Chen, Guo-Hong Yang

**Affiliations:** 1 Department of Traditional Chinese Medicine, Shanghai Institute of Pharmaceutical Industry, Shanghai 200040, China; 2 Department of Pharmacology, Institute of Medical Sciences, Shanghai Jiao Tong University School of Medicine, Shanghai 200025, China; 3 Department of Pharmacy, Shanghai Institute of Health Sciences, Shanghai 201318, China; 4 Department of Pharmacy, Health School Attached to Shanghai Jiao Tong University School of Medicine, Shanghai 201318, China; 5 Department of Pharmaceutical Sciences, School of Chemistry, Shanxi University, Taiyuan 030006, China

**Keywords:** *Peperomia dindygulensis* Miq., polyketides, antiangiogenic activity

## Abstract

Two new polyketides: 2*Z*-(heptadec-12-enyl)-4-hydroxy-3,4,7,8-tetrahydro-*2H*-chromen-5(*6H*)-one (**1**) and 2-(heptadec-12-enyl)-5-hydroxy-5,6,7,8-tetrahydrochromen- 4-one (**2**), together with eleven known compounds: 4-hydroxy-2-[(3,4-methylenedioxy- phenyl)tridecanoyl] cyclohexane-1,3-dione (**3**), oleiferinone (**4**), 4-hydroxy-2-[(3,4- methylenedioxyphenyl)undecanoyl]cyclohexane-1,3-dione (**5**), 4-hydroxy-2-[(11-phenyl- undecanoyl)cyclohexane-1,3-dione (**6**), proctorione C (**7**), surinone C (**8**), 5-hydroxy- 7,8,4'-trimethoxyflavone (**9**), 5-hydroxy-7,8,3',4'-tetramethoxyflavone (**10**), 5-hydroxy- 7,3',4'-trimethoxyflavone (**11**), 5,8-dihydroxy-7,3',4'-trimethoxyflavone (**12**) and cepharanone B (**13**) were isolated from the whole plant of *Peperomia dindygulensis* Miq. Their structures were elucidated by spectroscopic methods, including 2D-NMR techniques. Compounds **2**, **3**, **5** and **8** inhibited human umbilical vein endothelial cell (HUVEC) proliferation and compounds **5** and **8** sharply suppressed HUVEC tube formation.

## 1. Introduction

**Figure 1:**
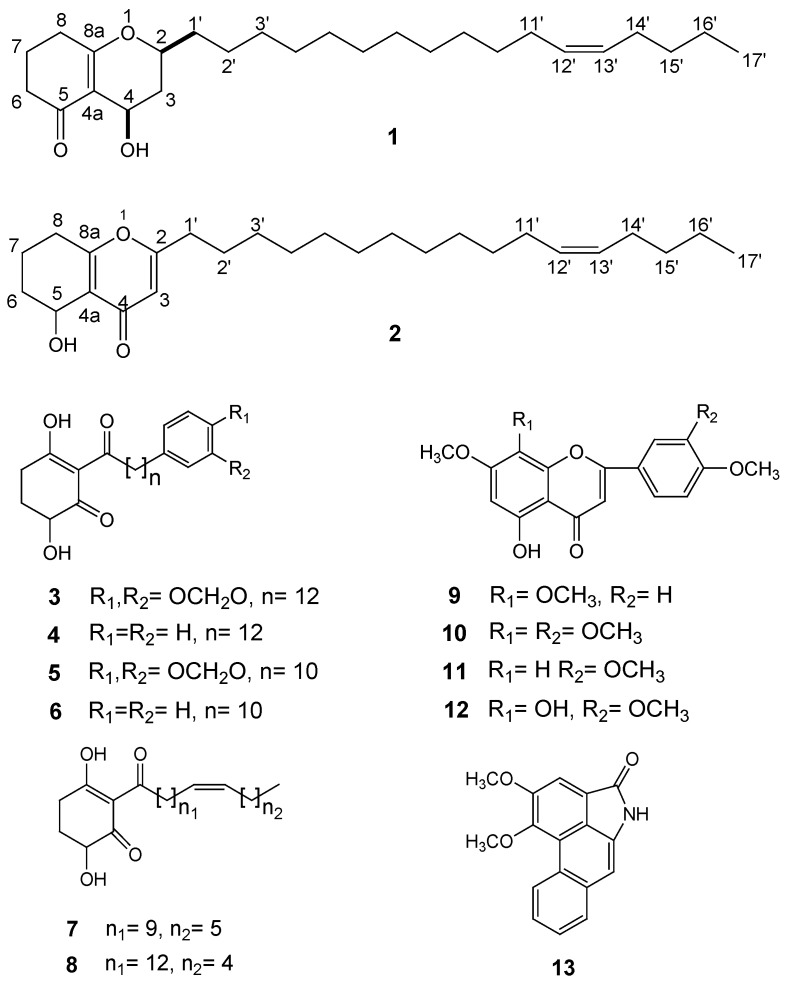
Structures of compounds **1**–**13**.

*Peperomia dindygulensis* Miq. (Piperaceae), a widespread herb in the south of China, is used in the folk medicine to treat cough, asthma, phthisis, and stomach, lung, mammary and liver cancers [1]. The common constituents of *Peperomia* genus include secolignans, tetrahydrofuran lignans, chromones and acylcyclohexane-1,3-diones [2,3,4,5,6,7], which possess various bioactivities, such as antitumor and anti-HIV activities [8]. We found that the chloroform extract prepared from *P. dindygulensis* showed significant suppression acitivity against the proliferation of primary human umbilical vein endothelial cells (HUVEC). In our previous research, we isolated some antiangiogenic secolignans including two new secolignans from *P. dindygulensis* [9]. Further fractionation and purification of the CHCl_3_ extract led to the isolation and characterization of two new polyketides: 2-(heptadec-12-enyl)-4-hydroxy-3,4,7,8- tetrahydro-*2H*-chromen-5(*6H*)-one (**1**) and 2-(heptadec-12-enyl)-5-hydroxy-5,6,7,8-tetrahydrochromen-4- one (**2**), together with eleven known compounds: 4-hydroxy-2-[(3,4-methylenedioxyphenyl)tridecanoyl] cyclohexane-1,3-dione (**3**) [10], oleiferinone (**4**) [11], 4-hydroxy-2-[(3,4-methylenedioxyphenyl) undecanoyl]cyclohexane-1,3-dione (**5**) [10], 4-hydroxy-2-[(11-phenylundecanoyl)cyclohexane-1,3-dione (**6**) [12], proctorione C (**7**) [13], surinone C (**8**) [14], 5-hydroxy-7,8,4'-trimethoxyflavone (**9**) [15], 5-hydroxy-7,8,3',4'-tetramethoxyflavone (**10**) [16], 5-hydroxy-7,3',4'-trimethoxyflavone (**11**) [17], 5,8-dihydroxy-7,3',4'-trimethoxyflavone (**12**) [15] and cepharanone B (**13**) [18] (Figure 1).

## 2. Results and Discussion

**Figure 2:**
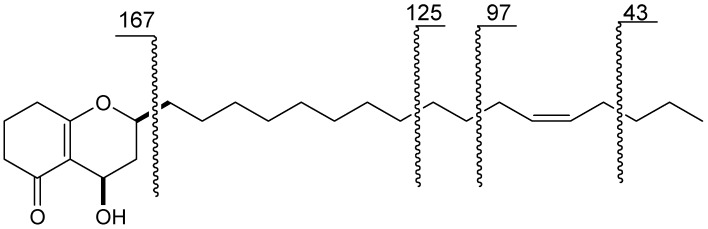
MS fragmentation of **1**.

**Figure 3:**
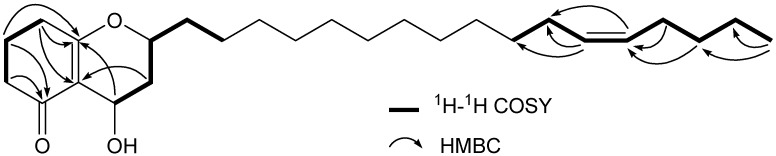
Key ^1^H-^1^H COSY and HMBC correlations of **1**.

Compound **1** was assigned the molecular formula C_26_H_44_O_3_ from the HRESI-MS *m/z *427.3185 [M+Na]^+^ peak (calcd for C_26_H_44_O_3_Na, 427.3188). The IR spectrum exhibited a hydroxyl absorption at 3,466 cm^−1^. A long alkyl chain was indicated from the multiple-proton signal in the range *δ*_H_ 1.26–1.37 in the ^1^H-NMR spectrum (Table 1). Two olefinic signals at *δ*_C_ 129.8 and 129.9 in the ^13^C-NMR spectrum (Table 1) suggested the presence of a double bond, and its position at C-12' was further confirmed by EI-MS (Figure 2), HMBC and ^1^H-^1^H COSY spectra (Figure 3). The *cis*-geometry of a double bond could be deduced from chemical shifts of C-11' (*δ*_C_ 27.2) and C-14' (*δ*_C_ 26.9) [19]. Two oxymethine [*δ*_H_ 4.00 (1H, *dddd*, 1.7, 5.4, 7.3,11.2, H-2) and *δ*_H_ 4.75 (1H, *dd*, 7.0, 9.3, H-4)] and five methylenes [*δ*_H_ 2.22 (1H, *ddd*, 2.0, 6.7, 13.5, H-3a) and 1.70 (1H, *m*, H-3b), *δ*_H_ 2.33 (1H, *dd*, 9.6, 16.8, H-6a) and 2.39 (1H, *m*, H-6b), *δ*_H_ 1.97 (1H, *dd*, *J *= 6.3, 12.9, H-7a) and 1.96 (1H, *dd*, *J *= 6.1, 12.9, H-7b), *δ*_H_ 2.40 (2H, *m*, H-8), *δ*_H_ 1.62 (1H, *m*, H-1'a) and 1.73 (1H, *m*, H-1'b)] were found to be connected to two moieties, ^6^CH_2_–^7^CH_2_–^8^CH_2_ and O–^4^CH–^3^CH_2_–^2^CH(O)–^1'^CH_2_ using the ^1^H–^1^H COSY spectrum. The above two moieties were linked by the two quaternary olefinic carbons *δ*_C_ 173.4 (C-8a) and 114.7 (C-4a) which showed HMBC cross-peaks with H-4, H-7, H-8, and with H-3, H-8, respectively. The location of carbonyl carbon *δ*_C_ 200.6 (C-5) could be confirmed by the HMBC correlations with H-6 and H-7. Thus, the moiety were determined as O=^5^C–^6^CH_2_–^7^CH_2_–^8^CH_2_–^8a^C=^4a^C–^4^CH(O)–^3^CH_2_–^2^CH(O)–^1'^CH_2_ and further confirmed by the homoallylic coupling between H-4 and H-8 in the ^1^H-^1^H COSY spectrum. The downfield chemical shift of C-8a and the remaining two degrees of unsaturation suggested that the oxygen at C-2 connected to C-8a, and C-5 to C-4a. The 5-hydroxytetrahydrochromen moiety was also confirmed by the mass spectrum which showed a fragment at *m/z *167 (Figure 2). Thus, the structure of **1** was elucidated as 2Z-(heptadec-12-enyl)-4-hydroxy-3,4,7,8-tetrahydro-*2H*-chromen-5(*6H*)-one. The NOE correlation between H-2 and H-4 indicated their *cis*-configuration.

**Table 1:** ^1^H- (400 MHz) and ^13^C-NMR (100 MHz) data of compound **1** (in CDCl_3_; *δ* in ppm, *J* in Hz).

Position	δ_C_	δ_H_
2	77.1	4.00 (1H, *dddd*, 1.7, 5.4, 7.3,11.2)
3	35.2	2.22 (1H, *ddd*,2.0, 6.7, 13.5); 1.70 (1H, *m*)
4	62.1	4.75 (1H, *dd*, 7.0, 9.3)
4a	114.7	
5	200.6	
6	36.7	2.33 (1H, *dd*, 9.6, 16.8); 2.39 (1H, *m*)
7	20.5	1.97 (1H, *dd*, 6.3, 12.9); 1.96 (1H, *dd*, 6.1, 12.9)
8	28.4	2.40 (2H, *m*)
8a	173.4	
1'	34.7	1.62 (1H, *m*); 1.73 (1H, *m*)
2'	25.0	1.46 (2H, *m*)
3'-10'	29.3–29.8	1.26–1.37 (16H, *m*)
11'	27.2	2.00 (2H, *m*)
12', 13'	129.8, 129.9	5.35 (2H, *m*)
14'	26.9	2.01 (2H, *m*)
15'	32.0	1.30 (2H, *m*)
16'	22.4	1.31 (2H, *m*)
17'	14.0	0.88 (3H, *t*, 7.0)
OH-4		4.63 ( *brs*)

**Figure 4:**
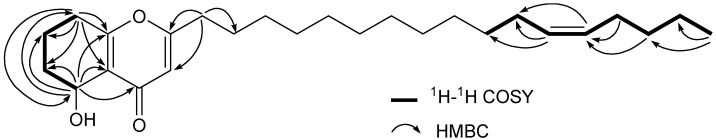
Key ^1^H-^1^H COSY and HMBC correlations of **2**.

Compound **2** had the molecular formula C_26_H_42_O_3_ as deduced from the HRESI-MS peak at *m/z *425.3029 [M+Na]^+^ (calcd for C_26_H_42_O_3_Na, 425.3032). The UV maxima at 249 nm, and the IR absorptions at 1,661, 1,605 cm^−1^ suggested the presence of a γ-pyrone ring, which was 2,3,5-trisubstituted according to the olefinic signals at *δ*_C_ 112.7 (C-3) and *δ*_H_ 6.10 (1H, s, H-3) in the NMR spectra (Table 2) [20]. Similar to compound **1**, it showed evidence for one moiety, ^5^CH(O)–^6^CH_2_–^7^CH_2_–^8^CH_2_, and a alkyl chain from the ^1^H-NMR, ^13^C-NMR, and ^1^H-^1^H COSY and HMBC spectra (Figure 4). In the HMBC spectrum, the proton of the oxymethylene *δ*_H_ 4.91 (1H, *br t*, *J *= 3.8 Hz, H-5) showed correlation with the carbonyl carbon (*δ*_C_ 180.7), C-4a (*δ*_C_ 123.2), C-6 (*δ*_C_ 29.5), C-7 (*δ*_C_ 18.1) and C-8 (*δ*_C_ 27.6), indicating that C-5 is connected with C-4a. The protons of the methylene [*δ*_H_ 2.46 (1H, *m*, H-8a) and 1.98 (1H, *m*, H-8b)] gave cross peaks with the carbons at C-5, C-6, C-7, C-4a and C-8a, but not with C-4, suggesting that C-8 is attached to C-8a. Surprisingly, the weak four bond reciprocal H-5/C-8 and H-8/C-5 HMBC correlations signals were observed. Although these may be considered unusual, such long-range correlations have been reported, especially in constrained ring systems [21,22,23]. The HMBC spectrum also suggested the linkage of C-2 and the alkyl chain. Thus, the structure of **2** was determined to be 2-(heptadec-12-enyl)-5-hydroxy-5,6,7,8- tetrahydrochromen-4-one.

**Table 2:** ^1^H- (400 MHz) and ^13^C-NMR (100 MHz) data of compound **2 **(in CDCl_3_; *δ* in ppm, *J* in Hz).

Position	δ_C_	δ_H_
2	169.3	
3	112.7	6.10 (1H, *s*)
4	180.7	
4a	123.2	
5	63.8	4.91 (1H, *br t*, 3.8)
6	29.5	1.76 (1H, *m*); 1.96 (1H, *m*)
7	18.1	1.74 (1H, *m*); 1.97 (1H, *m*)
8	27.6	2.49 (1H, *m*); 2.57 (1H, *m*)
8a	165.0	
1'	33.5	2.48 (2H, *t*, 7.6)
2'	26.8	1.61 (2H, *m*)
3'–10'	28.9–29.7	1.26–1.37 (16H, *m*)
11'	27.1	2.00 (2H, *m*)
12', 13'	129.8	5.35 (2H, *m*)
14'	26.9	2.01 (2H, *m*)
15'	31.9	1.30 (2H, *m*)
16'	22.3	1.31 (2H, *m*)
17'	14.0	0.89 (3H, *t*, 6.4)
5-OH		4.43 ( *br*s)

The structures of known compounds **3**~**13** were confirmed by detailed NMR and MS data comparison with those in the literature [10,11,12,13,14,15,16,17,18]. In addition, some similar compounds have been reported from *Trichoderma* [24,25].

**Figure 5:**
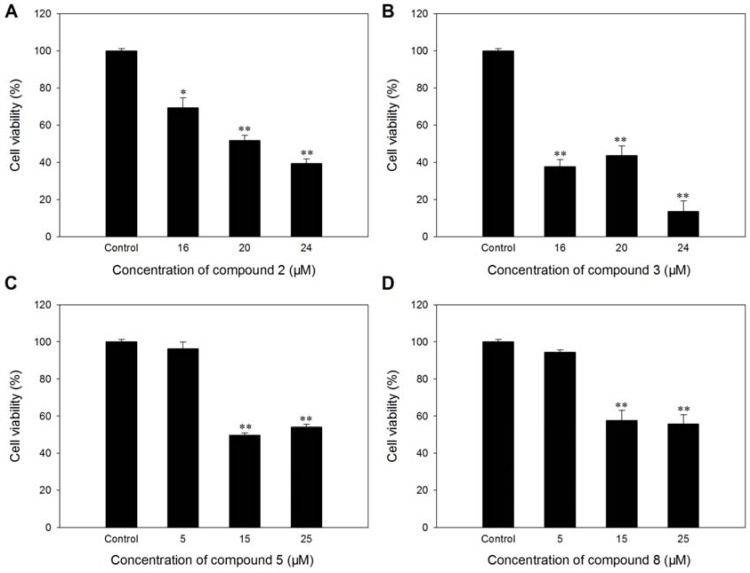
Effect of compounds **2** (**A**), **3** (**B**), **5** (**C**), **8** (**D**) on the viability of HUVEC after 48 h incubation. For blank control, the DMSO concentration was adjusted to below 0.1%. Values are expressed as mean ± SD, n = 4–6. * *p* < 0.05, ** *p* < 0.01 as compared with control.

**Figure 6:**
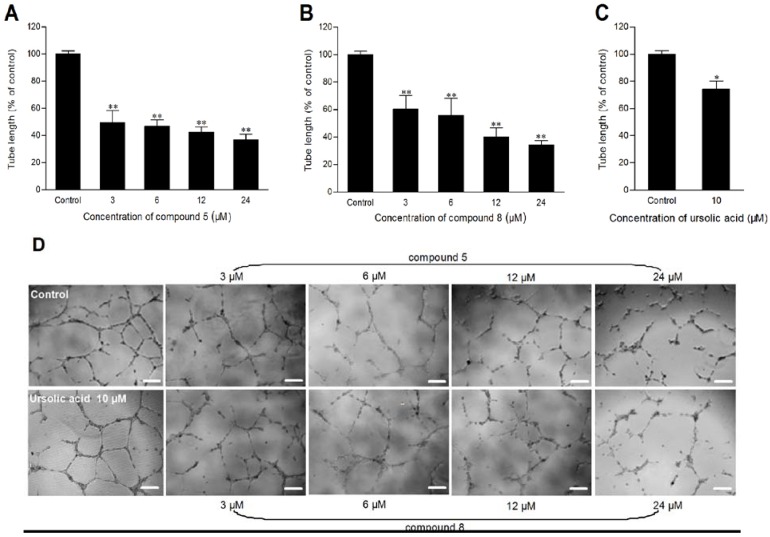
Effect of compounds **5**, compound **8**, and ursolic acid on HUVEC tube formation. **A**, **B**, and **C**: Tube length (% of control) after treatment with compound **5**, compound **8**, and ursolic acid (positive control), respectively. Values are expressed as mean ± SD, n = 4. * *p *< 0.05, ** *p *< 0.01 as compared with control. (**D**): The representative photographs of tube networks after treatment with ursolic acid (10 μM) and compounds **5** and **8** at various concentrations. Bar = 200 μm.

*In vitro* cytotoxicity of all the isolated compounds to HUVEC was examined after 48 h incubation. It was found that compounds **2**, **3**, **5**, **8** could dose dependently induced significant toxicity to HUVEC at different concentrations (Figure 5). The growth of HUVEC was almost completely inhibited by compound **3** at 24 μM. Compounds **5** and **8** also exhibited significant tube formation-inhibiting activity at 3, 6, 12 and 24 μM, respectively (Figure 6).

## 3. Experimental

### 3.1. General

Optical rotations were measured on a Perkin-Elmer 341 polarimeter. UV spectra were recorded on a Shimadzu UV-2500 PC spectrophotometer. IR spectra were recorded on a Nexus 670 spectrometer. NMR spectra were measured on a Bruker DRX 400 spectrometer and Bruker AV 500 spectrometer. EI-MS (70 eV) was carried out on an Autospec Premier P708 mass spectrometer. ESI-MS was carried out on a Waters Q-Tof micro YA019 mass spectrometer. Silica gel (200–300 mesh) was used for column chromatography, and pre-coated silica gel GF254 plates (Qingdao Marin Chemical Plant, Qingdao, China) were used for TLC.

### 3.2. Plant Material

*Peperomia dindygulensis* Miq., collected in Simao Region Yunnan Province of China in June 2008, was identified by Associate Professor Guo-Hong Yang. A voucher specimen (GHY-PDM20080728) has been deposited in the Herbarium of the Laboratory of Department of Traditional Chinese Medicine, Shanghai Institute of Pharmaceutical Industry, China.

### 3.3. Extraction and Isolation

The air-dried whole plant (10 kg) of *P. dindygulensis *was extracted exhaustively with 95% EtOH at r.t. (250 L, 7 days). The EtOH extract was evaporated *in vacuo* to yield a semisolid (1,300 g), of which 1,290 g was suspended in H_2_O (2,000 mL) and partitioned with CHCl_3_ (2,000 mL × 5) to yield 756 g of extract after concentration. Part of the CHCl_3_ extract (300 g) was subjected to column chromatography on Si gel (200~300 mesh, 3 kg) eluted with petroleum ether/EtOAc (100:0, 99:1, 49:1, 19:1, 9:1, 4:1, 7:3, 3:2, 1:1) and EtOAc to yield ten fractions (Frs. **1**–**10**). Fraction **6** (13.9 g) was separated repeatedly on silica gel columns (5 × 50 cm) using *n*-hexane/acetone (5:1) as eluent to obtain six fractions (**6A**–**F**). Fraction **6B** (227 mg) was further purified by Sephadex LH-20 and eluted with CHCl_3_/MeOH (1:1) to give **1** (10 mg). Fraction **6D** (139 mg) was purified repeatedly by Sephadex LH-20 using CHCl_3_/MeOH (1:1) as eluent to give **3** (20 mg). Fraction **7** (21.8 g) was separated repeatedly on silica gel columns (5 × 50 cm) using *n*-hexane/acetone (4:1) as eluent to obtain five fractions (**7A**–**E**). Fraction **7B** (1.6 g) was further purified by Sephadex LH-20 [eluted with CHCl_3_/MeOH (1:1)] to give four fractions, and the second fraction (700 mg) was applied to preparative silica gel TLC using *n*-hexane/acetone/acetic acid (5:1:0.1) as eluent to afford **4** (40 mg), **5** (100 mg) and **6** (12 mg). Fraction **7C** (7.6 g) was chromatographed over silica gel columns (3 × 50 cm) and eluted with *n*-hexane/EtOAc (3:1) to obtain five fractions, the second fraction (154 mg) was applied to preparative silica gel TLC using toluene/EtOAc (9:1) as eluent to give **11** (4 mg). Fraction **8** (4.5 g) was chromatographed over silica gel columns (3 × 50 cm) eluted with CHCl_3_/MeOH (50:1) to obtain four fractions (**8A**–**D**). Fraction **8B** (205 mg) was recrystallized from CHCl_3_/MeOH (1:1) to afford **9** (51 mg). Fraction **8C** (600 mg) was recrystallized from CHCl_3_/MeOH (1:1) to afford **10** (246 mg). Fraction **8D** (1.4 g) was chromatographed over silica gel columns (3 × 50 cm) and eluted with *n*-hexane/acetone/acetic acid (100:10:1) to give **2** (277 mg) and **6** (36 mg). Fraction **9** (18.9 g) was separated repeatedly on silica gel columns (5 × 50 cm) using n-hexane/acetone (4:1) as eluent to obtain eight fractions (**9A**–**H**). Fraction **9B** (1.5 g) was further separated repeatedly on silica columns (3 × 50 cm) and eluted with CHCl_3_/acetone (200:1) to give three fractions, and the second fraction was subjected to preparative silica gel TLC using *n*-hexane/acetone/acetic acid (100:10:1) as eluent to give **8** (20 mg). Fraction **9D** (1.8 g) was chromatographed on silica gel columns (3 × 50 cm) using *n*-hexane/acetone (5:1) as eluent to obtain three fractions, and the last fraction (222 mg) was further purified by Sephadex LH-20 and eluted with CHCl_3_/MeOH (1:1) to give **13** (5 mg). Fraction **9E** (12.2 g) was separated repeatedly on silica gel columns (5 × 50 cm) using CHCl_3_ as eluent to obtain six fractions, and the last fraction was separated repeatedly by Sephadex LH-20 and eluted with CHCl_3_/MeOH (1:1) to give **12** (12 mg).

*2Z-(Heptadec-12-enyl)-4-hydroxy-3*,*4*,*7*,*8-tetrahydro-2H-chromen-5(6H)-one* (**1**). Yellowish oil; [α]_D_^13^ 126° (c = 0.368, CHCl_3_); UV λ_max_ (MeOH) nm (log ε): 258 (4.44); IR (NaCl) ν_max_: 3466, 2925, 2854, 1613, 1427, 1370, 1249, 1188, 1083, 1054 cm^−1^; EI-MS *m/z *404 [M]^+^ (9.1), 386 (100.0), 167 (24.1), 139 (50.7), 125 (43.1), 111(41.9), 97 (5.2), 43 (21.5); ^1^H- and ^13^C-NMR data: see Table 1; HRESI-MS *m/z *427.3185([*M*+Na]^+^, calcd for C_26_H_44_O_3_Na 427.3188).

*2-(Heptadec-12-enyl)-5-hydroxy-5*,*6*,*7*,*8-tetrahydrochromen-4-one* (**2**). Yellowish oil; [α]_D_^13^ 34° (c = 0.435, CHCl_3_); UV λ_max_ (MeOH) nm (log ε): 216 (3.66), 249 (3.68); IR(NaCl) ν_max_: 3431, 3003, 2926, 2854, 1716, 1661, 1605, 1436, 1176, 1086, 950, 859, 722 cm^−1^; ^1^H- and ^13^C-NMR data: see Table 2; HRESI-MS *m/z* 425.3029 ([*M*+Na]^+^, calcd for C_26_H_42_O_3_Na 425.3032).

### 3.4. Antiangiogenic Activity Assays

The effect of isolated compounds on the proliferation of HUVEC was evaluated by CCK-8. HUVEC tube formation was conducted for the assay of *in vitro* angiogenesis using the Chemicon *in vitro* angiogenesis assay kit (ECM625). Details of the assays were provided in a previous report [9].

## 4. Conclusions

Two new polyketides: 2*Z*-(heptadec-12-enyl)-4-hydroxy-3,4,7,8-tetrahydro-*2H*-chromen-5(*6H*)-one (**1**), and 2-(heptadec-12-enyl)-5-hydroxy-5,6,7,8-tetrahydrochromen-4-one (**2**), were isolated from *Peperomia dindygulensis*, together with eleven known compounds **3**–**13**. Compounds **2**, **3**, **5** and **8** inhibited human umbilical vein endothelial cells (HUVEC) proliferation and compounds **5** and **8** sharply suppressed HUVEC tube formation.
